# Treatment outcome of elderly patients (≥78 years) with head and neck squamous cell carcinoma: A single center experience

**DOI:** 10.17305/bb.2024.10516

**Published:** 2024-10-01

**Authors:** Yi Liu, Dan Wang, Yue Deng, Shichuan Zhang

**Affiliations:** 1 Department of Oncology, Affiliated Hospital of Southwest Medical University, Luzhou, China; 2 Department of Radiation Oncology, Radiation Oncology Key Laboratory of Sichuan Province, Sichuan Clinical Research Center for Cancer, Sichuan Cancer Hospital and Institute, Sichuan Cancer Center, Affiliated Cancer Hospital of University of Electronic Science and Technology of China, Chengdu, China

**Keywords:** Elderly, head and neck cancer, treatment

## Abstract

Patients older than the expected age of the local population generally have limited life expectancy. The optimal treatment approach for very elderly patients with head and neck cancer remains uncertain. This study retrospectively analyzed patients over 78 years old, the expected age in 2019 for Chinese individuals, who underwent treatment for head and neck cancer at a tertiary cancer center in China. The study compared the overall survival rates among different treatment groups. The findings revealed that among patients eligible for surgery, radical resection yielded better outcomes compared to radiotherapy-based treatments, with a hazard ratio (HR) of 0.362 (95% confidence interval [CI] 0.160–0.819, *P* ═ 0.015). Among patients who received radiotherapy, those who received a total dose exceeding 60 Gy had a significantly longer survival compared to those who received palliative doses, with a median survival time of 31 months vs 14 months (*P* ═ 0.003). Among 78 patients who underwent conventional fractionated radiotherapy (CFRT), 15 patients (19.23%) experienced unscheduled treatment breaks with a median duration of 12 days. However, these treatment breaks did not appear to impact survival (*P* > 0.1). The study also suggested that altered fractionated radiotherapy, including hypofractionated radiotherapy (hypo-RT), could be a viable alternative to CFRT, offering similar survival outcomes with reduced treatment duration. In conclusion, eligible patients should be treated with curative intent, even if they are older than the expected age of the local population. When radiotherapy is indicated, altered fractionation, particularly hypo-RT, may be a favorable option to consider.

## Introduction

Clinicians often ponder whether elderly patients can withstand standard treatments, although patient age is not usually considered a determining factor for treatment options according to guidelines. This concern extends to patients and their families, as in the real world, a significant proportion of elderly patients do not strictly adhere to guideline-recommended treatments. This deviation may stem from physician adjustments or patient preferences [1, 2]. Notably, there is scarce high-level evidence to guide the treatment of elderly patients, particularly in the context of head and neck cancer, where functional impairment from both cancer progression and treatment-related side effects is more pronounced compared to tumors in other locations.

The term “elderly” defines individuals aged 65 years and older [3]. Within this broad age spectrum, variations in treatment outcomes may exist among different age subgroups. Several cohort studies have indicated that elderly patients can safely undergo definitive chemoradiotherapy and derive benefits from it [4]. However, for subgroups exceeding the age range of 78–80 in developed countries, the acceptability of such treatments may be diminished. For instance, a retrospective study on head and neck cancer using the Surveillance, Epidemiology, and End Results (SEER) database revealed that patients aged 80 and above had a significantly higher risk of mortality compared to those aged 66–69 years (hazard ratio [HR] 2.24, 95% confidence interval [CI] 2.17–2.31) [5]. Similarly, in a cohort of patients with locally advanced head and neck cancer from the National Cancer Database (NCDB) who underwent concurrent chemoradiotherapy, individuals older than 77 years had 1.76 times higher risk of death than those aged 71–76 years (95% CI 1.63–1.90) [6, 7]. Therefore, the optimal treatment approach for these older patients necessitates careful evaluation.

This study aims to investigate treatment outcomes of elderly patients at a single tertiary cancer center in China. All patients included in the study were aged over 78 years at the initiation of treatment, a threshold surpassing the expected age of 77.4 years in China as of 2019. The efficacy of different treatment modalities, including surgery, radiotherapy, and chemoradiotherapy is compared to offer real-world insights into the optimal management of geriatric patients with head and neck squamous cell carcinoma.

## Materials and methods

### Patients

**Figure 1.**
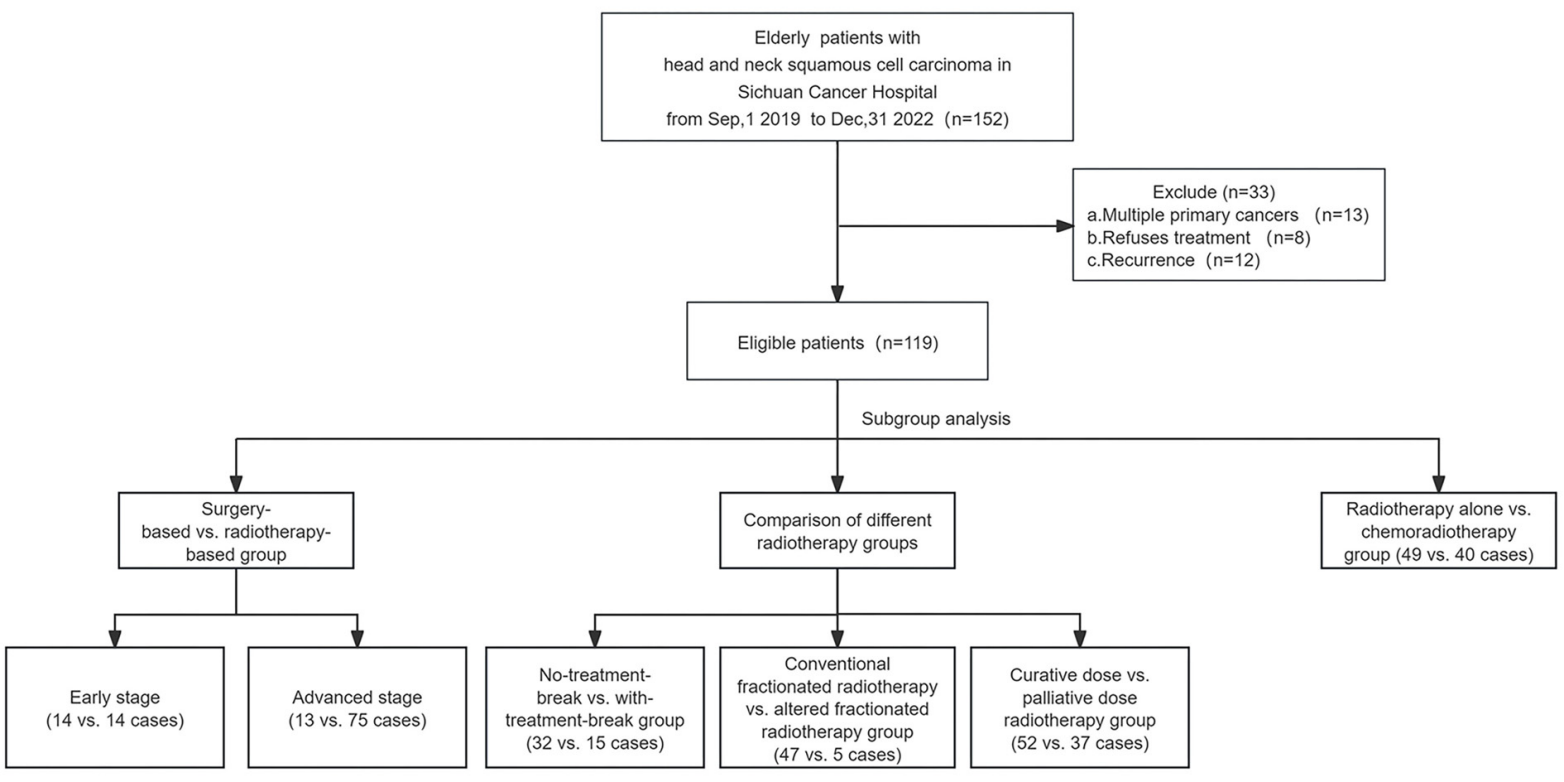
Study design and the workflow diagram.

The study was conducted retrospectively on patients who fulfilled the inclusion criteria. Inclusion criteria comprised patients aged 78 years with a performance score of 0–1, histologically confirmed squamous cell carcinoma in the head and neck, who received treatment between September 1, 2019, and December 31, 2022. Patients with recurrent tumors or synchronous second primary tumors were excluded (Figure 1). Tumor clinical staging was based on the 8^th^ edition of the American Joint Committee on Cancer (AJCC) staging system.

### Treatment

Treatment modalities included surgery alone, surgery followed by radiotherapy/chemoradiotherapy, radiotherapy alone, chemoradiotherapy, and chemotherapy alone. All radiation treatments utilized intensity-modulated radiotherapy. Chemotherapy regimens consisted of both single agents (cisplatin, carboplatin, nadaplatin, or capecitabine) and doublet agents (paclitaxel and cisplatin, carboplatin, nadaplatin, or 5-FU and cisplatin).

### Ethical statement

The study was approved by the Ethics Committee of Sichuan Cancer Hospital. All data were anonymized, and the requirement for individual consent for this retrospective analysis was waived.

### Statistical analysis

Overall survival was calculated from the start of the pathologic diagnosis to any cause of death. Survival analysis utilized the Kaplan–Meier curve and log-rank test. Spearman correlation was performed on all variables and was represented as a visual heat map. Univariate and multivariate Cox proportional hazard regressions were utilized to assess the relationship between variables and survival outcomes. Statistical analyses were conducted using SPSS software version 27 (IBM, Armonk, NY, USA), with a significance level set at *P* < 0.05 for all analyses.

## Results

### Patient characteristics

A total of 152 patients, with a minimum age of 78 years, underwent screening. Thirteen patients with a second primary tumor were excluded, while 12 patients were identified as having previously treated recurrent tumors. Additionally, eight patients declined any form of treatment. Finally, 119 patients were enrolled in the study. The characteristics of included patients are detailed in Table 1. The male-to-female ratio was 2.5:1, with a median age of 81 years (range: 78–107).

**Table 1:** Clinical characteristics of the 119 elderly patients with head and neck squamous cell carcinoma

**Characteristic**	**N (%)**
*Sex*	
Male	85 (71.43)
Female	34 (28.57)
*Age (years)*	
Median (range)	81 (78–107)
*Primary site*	
Larynx	28 (23.53)
Oral cavity	63 (52.94)
Oropharynx	12 (10.09)
Hypopharynx	6 (5.04)
Skin of head and neck region	10 (8.40)
T category*	
1	12 (10.09)
2	25 (21.01)
3	46 (38.65)
4	34 (28.57)
Missing	2 (1.68)
N category*	
0	66 (55.46)
1	26 (21.85)
2	12 (10.09)
3	13 (10.92)
Missing	2 (1.68)
M category*	
0	115 (96.64)
1	4 (3.36)
*Clinical stage*	
I	11 (9.24)
II	17 (14.29)
III	43 (36.13)
IV	46 (38.66)
Missing	2 (1.68)
*Treatment*	
Radiotherapy alone	49 (41.18)
Chemoradiotherapy	40 (33.61)
Surgery followed by radiotherapy	16 (13.45)
Surgery alone	8 (6.72)
Surgery followed by chemoradiotherapy	5 (4.20)
Chemotherapy	1 (0.84)

*According to the 8th edition of the American Joint Committee on Cancer (AJCC) TNM staging.

More than half of the patients (52.94%) presented with oral cavity tumors. Other primary tumor sites included the larynx (23.53%), oropharynx (10.09%), skin (8.40%), and hypopharynx (5.04%). The majority of patients (74.79%) were diagnosed at an advanced stage (Stages III and IV). Notably, two patients who underwent surgery could not be staged due to inadequate pre-treatment imaging.

### Treatment characterization and outcome

Among the 119 enrolled patients, treatment strategies varied. One patient received chemotherapy alone, while the remaining patients were categorized into two main groups based on treatment modality: surgery-based and radiotherapy-based groups.

The surgery-based group comprised patients who underwent curative intent surgery with or without additional treatments. Subgroups within this category included surgery alone (8 cases, 6.72%), surgery followed by radiotherapy (16 cases, 13.45%), and surgery followed by chemoradiotherapy (5 cases, 4.20%).

Conversely, the radiotherapy-based group included patients who received radiotherapy as the primary treatment modality. This group was further divided into those who received radiotherapy alone (49 cases, 41.18%) and those who underwent chemoradiotherapy (40 cases, 33.61%).

In the radiotherapy-based group, the majority of patients (87.64%) received conventional fractionated radiotherapy (CFRT), with total doses ranging from 4.06 to 79.74 Gy. A small portion of patients (7.87%) received hypofractionated radiotherapy (hypo-RT), while a few (3.37%) underwent a combination of CFRT and late-course hyperfractionated radiotherapy (hyper-RT).

The coherence of fractionated radiotherapy was assessed, revealing that 39.74% of patients who received CFRT were administered palliative doses lower than 60 Gy. Among those who received curative doses, 58.43% completed the treatment regimen. Additionally, 16.85% of patients experienced treatment breaks exceeding five days.

For patients who received concurrent chemoradiotherapy, the regimens included cisplatin (10.00%), carboplatin (10.00%), nadaplatin (2.50%), a combination of cisplatin and nadaplatin (2.50%), and oral capecitabine (65.00%).

**Figure 2.**
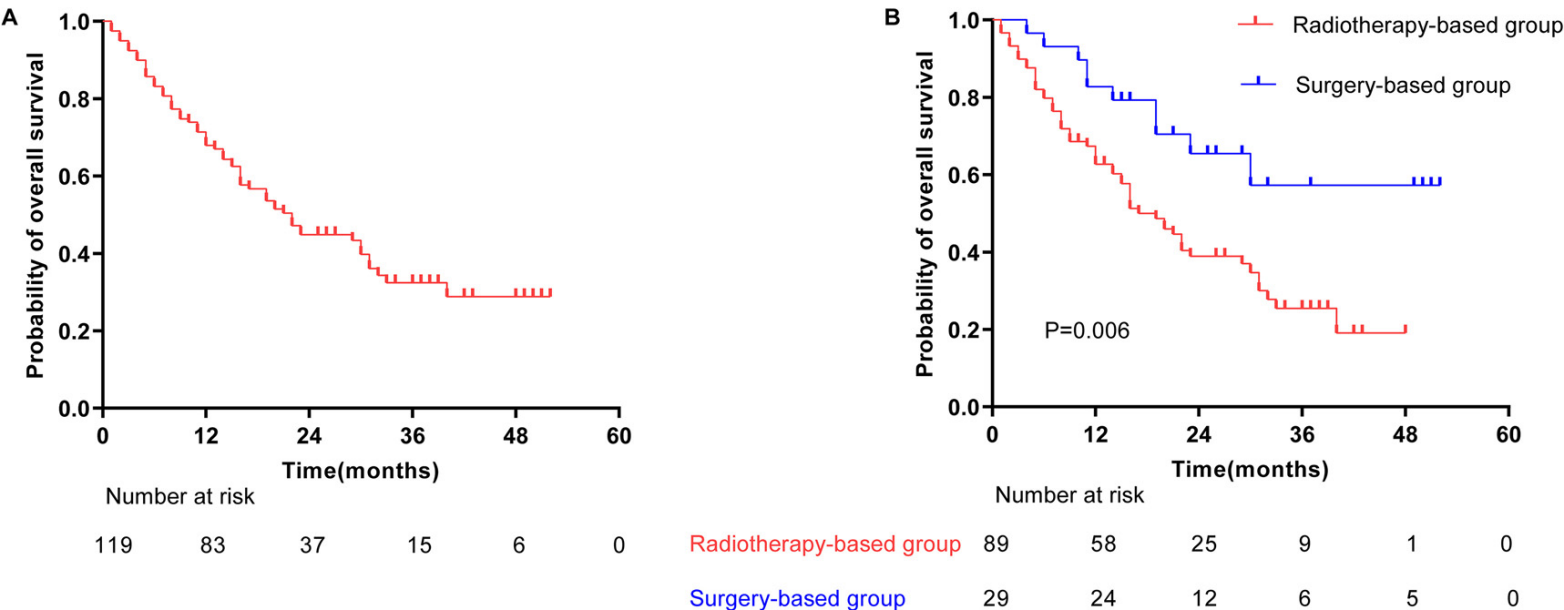
**Kaplan–Meier survival curves of overall survival.** (A) Overall survival for the 119 patients; (B) Overall survival for patients stratified into the surgery-based and radiotherapy-based group. *P* values were calculated using the log-rank test.

During the follow-up period, 57.98% of patients succumbed to the disease. Among those alive at the last follow-up, the median follow-up duration was 29 months (range: 10–52 months). The overall median survival for the cohort was 22 months, with 1-year, 2-year, and 3-year survival rates of 67.93%, 44.85%, and 32.41%, respectively (Figure 2A).

In the surgery-based group, the median survival had not been reached, with 1-year, 2-year, and 3-year survival rates of 82.76%, 65.46%, and 57.28%, respectively. Conversely, in the radiotherapy-based group, the median survival was 19 months, with corresponding 1-year, 2-year, and 3-year survival rates of 62.73%, 38.93%, and 25.43%, respectively (Figure 2B).

### Factors associated with overall survival

**Figure 3.**
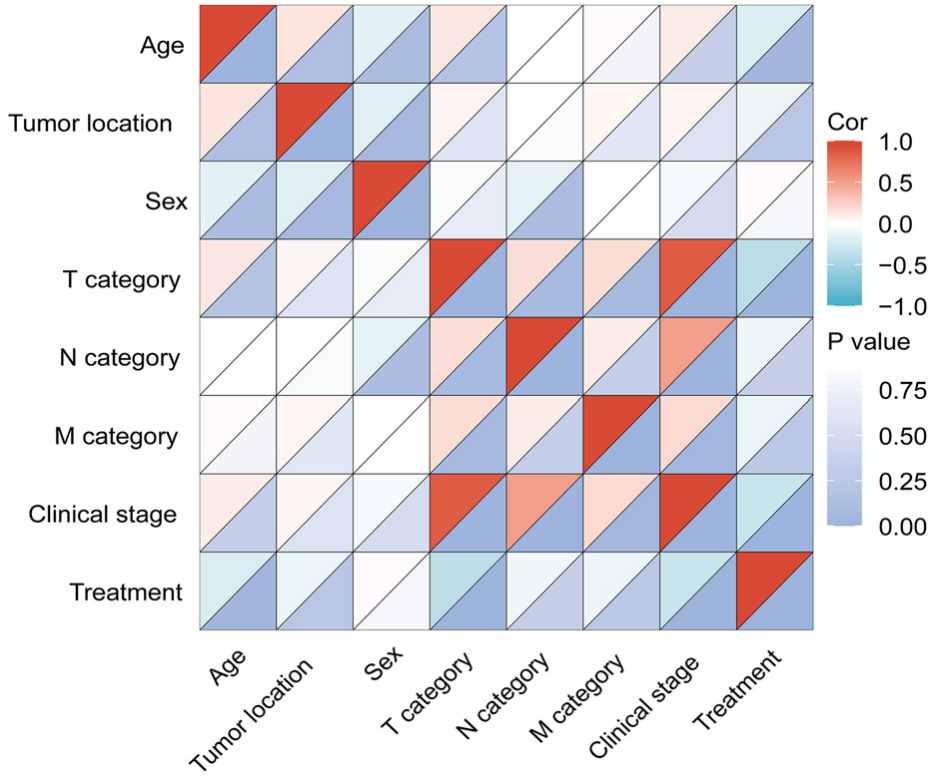
The correlation analysis of factors influencing the overall survival.

Firstly, when analyzing factors influencing overall survival, a significant correlation between the T/N/M category and the clinical stage was revealed (Figure 3).

**Figure 4.**
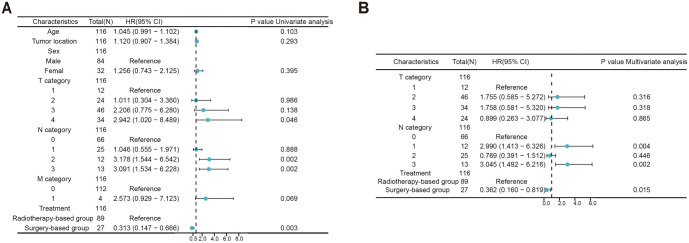
**Forest plots of (A) univariate analysis and (B) multivariate analysis depict the HR and 95% CI for overall survival by subgroup.** HR: Hazard ratio; CI: Confidence interval.

Given that the clinical stage is dictated by the TNM classification, the clinical stage was excluded in the subsequent analysis. It was found that T category, N category, and treatment modality were significant in univariate analysis (Figure 4A). However, in the multivariate analysis, only N category and treatment modality were proved as independent factors affecting treatment outcomes (Figure 4B). Notably, patients who received surgery-based treatment have significantly reduced death risk compared with those who received radiotherapy-based treatment [HR 0.362 (95% CI 0.160–0.819), *P* ═ 0.015].

### Subgroup analysis

#### Surgery-based vs radiotherapy group in early-stage diseases (stages I and II)

**Figure 5.**
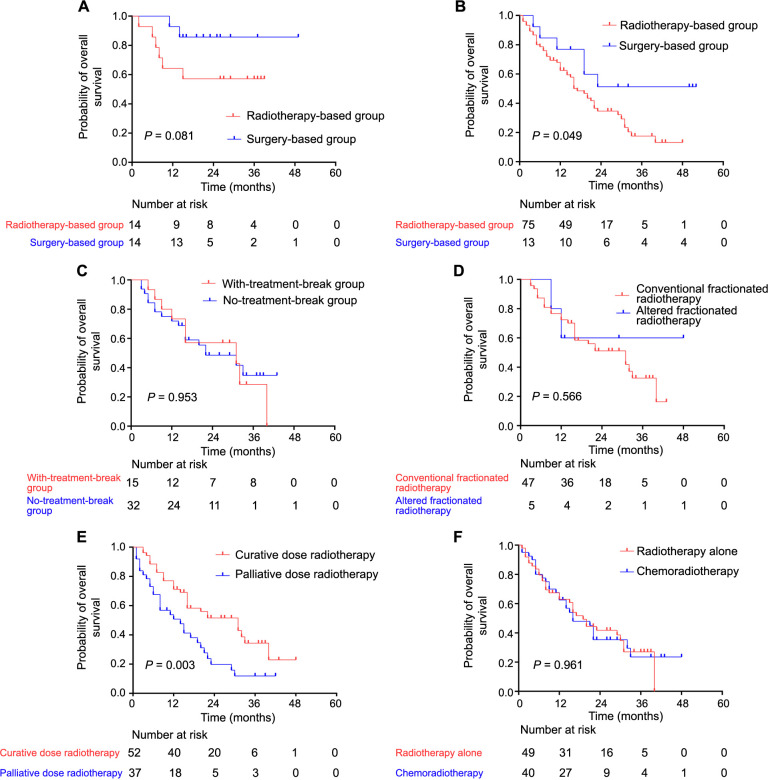
**Kaplan–Meier survival curves of overall survival.** (A) Overall survival for patients stratified into the surgery-based and radiotherapy group with the early stage diseases (stages I and II); (B) Overall survival for patients stratified into the surgery-based and radiotherapy-based group with advanced stage (stages III and IV); (C) Overall survival for patients stratified into the no-treatment-break group and with-treatment-break group in 47 patients receiving curative dose; (D) Overall survival for patients stratified into the conventional fractionated radiotherapy and altered fractionated radiotherapy group in 52 patients receiving curative dose; (E) Overall survival for patients stratified into the curative dose radiotherapy and palliative dose radiotherapy group in 89 patients receiving radiotherapy-based treatment; (F) Overall survival for patients stratified into the radiotherapy alone and chemoradiotherapy group in 89 patients receiving radiotherapy-based treatment. *P* values were calculated using the log-rank test.

A comparison of survival outcomes between patients receiving surgery-based and radiotherapy-based treatments for early-stage diseases (stages I and II) revealed that 14 out of 28 patients underwent surgery, while the other 14 received radiotherapy alone. There was no significant difference in clinical characteristics between the two groups (Table 2). At the last follow-up, two patients in the surgery-based group and six in the radiotherapy group died. Although the survival trend favored the surgery-based group, the difference did not reach statistical significance (*P* ═ 0.081) (Figure 5A).

#### Comparison of surgery-based and radiotherapy-based groups in advanced stages (stages III and IV)

In the advanced stages (stages III and IV), 13 patients underwent surgery-based therapy, while 75 received radiotherapy-based therapy. Patients who received surgery were significantly younger and had small primary tumors (early T categories) compared to those in the radiotherapy group (Table 2). The 1-year, 2-year, and 3-year survival rates for the two groups were 62%, 40%, 34% and 63%, 17%, 54%, respectively. The results indicated that surgery-based treatment continued to yield better survival outcomes than radiotherapy-based treatment (*P* ═ 0.049) (Figure 5B).

#### Impact of interruption of radiotherapy on survival

Among the 78 patients who received CFRT, 47 received a curative dose of 60 Gy. Fifteen of them experienced a non-scheduled treatment break of over five days between fractions due to treatment toxicity, with a median break duration of 12 days. However, the treatment breaks did not appear to negatively impact survival (*P* > 0.1) (Figure 5C).

#### Comparison of CFRT and altered fractionated radiotherapy

Among the 89 patients who received radiotherapy-based treatment, 11 underwent altered fractionated radiotherapy, including hypo-RT and hyper-RT. Five of these patients received a curative dose equivalent to 60 Gy (equivalent dose in 2 Gy, EQD2, a/β ═ 10). Survival analysis revealed that these patients had comparable outcomes to those who received a curative dose of CFRT (*P* > 0.1) (Figure 5D).

#### Comparison of survival between curative dose and palliative dose radiotherapy

Out of the 89 patients who received radiotherapy, 52 were treated with a curative dose while 37 received a palliative dose, irrespective of conventional or altered fractionation. A significant survival benefit was observed in patients receiving curative dose radiotherapy compared to those receiving palliative doses (median survival: 31 vs 14 months, *P* ═ 0.003) (Figure 5E).

#### Comparison of radiotherapy alone and chemoradiotherapy

Among the 89 patients who underwent radiotherapy-based treatment, 49 received radiotherapy alone, while 40 received chemoradiotherapy. No significant difference in survival outcomes was observed between these two groups (Figure 5F).

## Discussion

Generally, patients exceeding the expected age threshold may have limited life expectancies, raising questions about the need for more palliative approaches to their treatment. The current study demonstrated that for head and neck cancer, radical resection for these patients yielded superior survival outcomes compared to non-surgical treatments (HR: 0.362, 95% CI 0.160–0.819, *P* ═ 0.015). However, it is worth mentioning that surgery was more commonly recommended for relatively younger patients and those with early-stage disease. There were no patients over 86 years who underwent surgical procedures.

**Table 2:** Baseline of clinical characteristics in surgery-based and radiotherapy-based groups in early-stage diseases (stages I and II) and advanced stages (stages III and IV)

**Characteristic**	**Stages I and II**				**Stages III and IV**			
	**Total (*n* ═ 28)**	**Radiotherapy-based group (*n* ═ 14)**	**Surgery-based group (*n* ═ 14)**	***P* value**	**Total (*n* ═ 88)**	**Radiotherapy-based group (*n* ═ 75)**	**Surgery-based group (*n* ═ 13)**	***P* value**
*Age (years)*				0.609				0.006
Median (range)	81 (78–90)	81 (78–90)	80.5 (78–86)		81 (78–107)	81 (78–107)	79 (78–86)	
*Sex*				1				1
Male	22 (78.57)	11 (78.57)	11 (78.57)		62 (70.45)	53 (70.67)	9 (69.23)	
Female	6 (21.43)	3 (21.43)	3 (21.43)		26 (29.55)	22 (29.33)	4 (30.77)	
*Primary site*				0.155				0.735
Larynx	8 (28.57)	6 (42.86)	2 (14.29)		20 (22.73)	16 (21.33)	4 (30.77)	
Oral cavity	14 (50.00)	5 (35.71)	9 (64.29)		46 (52.27)	39 (52.00)	7 (53.85)	
Oropharynx	2 (7.14)	0 (0.00)	2 (14.29)		10 (11.36)	8 (10.67)	2 (15.38)	
Hypopharynx	3 (10.71)	2 (14.29)	1 (7.14)		3 (3.41)	3 (4.00)	0 (0.00)	
Skin of head and neck	1 (3.57)	1 (7.14)	0 (0.00)		9 (10.23)	9 (12.00)	0 (0.00)	
*T category*				1				0.014
1	11 (39.29)	6 (42.86)	5 (35.71)		1 (1.14)	0 (0.00)	1 (7.69)	
2	16 (57.14)	8 (57.14)	8 (57.14)		8 (9.09)	5 (6.67)	3 (23.08)	
3	1 (3.57)	0 (0.00)	1 (7.14)		45 (51.14)	38 (50.67)	7 (53.85)	
4					34 (38.64)	32 (42.67)	2 (15.38)	
*N category*								0.298
0	28 (100.00)	14 (100.00)	14 (100.00)		38 (43.18)	32 (42.67)	6 (46.15)	
1					25 (28.41)	23 (30.67)	2 (15.38)	
2					12 (13.64)	11 (14.67)	1 (7.69)	
3					13 (14.77)	9 (12.00)	4 (30.77)	
*M category*								1
0	28 (100.00)	14 (100.00)	14 (100.00)		84 (95.45)	71 (94.67)	13 (100.00)	
1					4 (4.55)	4 (5.33)	0 (0.00)	

Numbers are represented and *n* (%) or median (range).

For patients in advanced stages, the survival rates post radiotherapy or chemoradiotherapy appeared less favorable, with a median survival of 17 months. The Kaplan–Meier survival curves exhibited a rapid decline over time compared to the surgery group. Further analysis within the radiotherapy subgroup revealed that a curative dose equivalent to 60 Gy or more was associated with significantly improved survival (*P* ═ 0.003). Notably, treatment interruptions during fractionated radiation did not negatively impact final outcomes, suggesting that achieving a curative dose is crucial. In cases of severe acute toxicity, a one- to two-week break in treatment can facilitate patient recovery without compromising radiotherapy efficacy. Nonetheless, it is important to note that the current study has relatively short follow-up, and the long-term implications of treatment interruptions on survival need further investigation.

While chemoradiotherapy is the standard approach for advanced head and neck cancer, previous studies have indicated that adding chemotherapy to radiotherapy may not be suitable for very elderly patients. Our study corroborates this finding when comparing survival outcomes between radiotherapy alone and chemoradiotherapy groups.

CFRT with one daily fraction totaling 30–35 fractions over six weeks may pose significant challenges for elderly patients with limited life expectancies. In such cases, hypo-RT, using larger doses per fraction and fewer fractions, presents a viable alternative to CFRT.

Various hypo-RT regimens, such as the QUAD SHOT regimen (3.7 Gy twice daily for two days every three weeks for three cycles) [8], SCAHRT regimen (60 Gy in 20 fractions with a 2–4 week break) [9], and weekly regimen (56–64 Gy in 7–8 fractions once a week) [10], have been proposed as alternatives to CFRT. In our study, seven patients received hypo-RT with fraction doses ranging from 3.90 to 5.05 Gy, demonstrating comparable survival outcomes to those who underwent CFRT.

The present study has its limitations. The study was conducted at a single center with a relatively small patient population. The conclusions require further validation in multi-centered, prospective studies. Some important factors, including performance status and comorbidities, were not discussed in the current study as patients enrolled in this study were all scored as 0–1 for ECOG score, and records for comorbidity were incomplete for most patients treated in outpatient clinics. The absence of toxicity records precluded an assessment of treatment impact on patient quality of life, a crucial factor in treatment decision-making.

## Conclusion

This retrospective analysis provides valuable real-world insights into the management of head and neck squamous cell carcinoma in very elderly patients. The findings emphasize the effectiveness of radical resection or curative-dose radiotherapy (>60 Gy) for eligible individuals. Nevertheless, the overall survival of the cohort remains modest at 22 months, highlighting the urgent need for improved treatment approaches for this unique patient population.
